# Crystal structure of μ-1κ*C*:2(η^2^)-carbonyl-carbonyl-1κ*C*-chlorido-2κ*Cl*-μ-chlorido­borylene-1:2κ^2^ *B*:*B*-[1(η^5^)-penta­methyl­cyclo­penta­dien­yl](tri­cyclo­hexyl­phosphane-2κ*P*)iron(II)platinum(II) benzene monosolvate

**DOI:** 10.1107/S1600536814023381

**Published:** 2014-10-29

**Authors:** Holger Braunschweig, Thomas Kramer

**Affiliations:** a Institut für Anorganische Chemie, Universität Würzburg, Am Hubland, D-97074 Würzburg, Germany

**Keywords:** crystal structure, heterodinuclear compound, borylene, platinum, oxidative addition

## Abstract

In the mol­ecular structure of the dinuclear title compound [η^5^-(C_5_(CH_3_)_5_)(CO)Fe{(μ-BCl)(μ-CO)}PtCl(P(C_6_H_11_)_3_)]·C_6_H_6_, the two metal atoms, iron(II) and platinum(II), are bridged by one carbonyl (μ-CO) and one chlorido­borylene ligand (μ-BCl). The Pt^II^ atom is additionally bound to a chloride ligand situated *trans* to the bridging borylene, and a tri­cyclo­hexyl­phosphane ligand (PCy_3_) *trans* to the carbonyl ligand, forming a distorted square-planar structural motif at the Pt^II^ atom. The Fe^II^ atom is bound to a penta­methyl­cyclo­penta­dienyl ligand [η^5^-C_5_(CH_3_)_5_] and one carbonyl ligand (CO), forming a piano-stool structure. Additionally, one benzene solvent mol­ecule is incorporated into the crystal structure, positioned staggered relative to the penta­methyl­cyclo­penta­dienyl ligand at the Fe^II^ atom, with a centroid–centroid separation of 3.630 (2) Å.

## Abstract 2

The title compound [η^5^-(C_5_(CH_3_)_5_)(CO)Fe{(μ-BCl)(μ-CO)}PtCl(P(C_6_H_11_)_3_)]·C_6_H_6_ shows a piano-stool coordination geometry at the Fe^II^ atom and a distorted square-planar coordination geometry at the Pt atom. Both metals are bridged by one carbonyl and one chlorido­borylene unit. Additionally, one benzene solvent mol­ecule aligns in a staggered position relative to the (penta­meth­yl)cyclo­penta­dienyl ligand of the Fe^II^ centre.

## Chemical context   

In 2005, Braunschweig *et al.* reported the compound [(η^5^-C_5_Me_5_)Fe(μ-BCl_2_)(μ-CO)_2_Pd(PCy_3_)] (Me is methyl and Cy is cyclo­hex­yl) with a novel bonding motif featuring a BCl_2_ unit bridging an Fe and an Pd atom. This compound was isolated upon the reaction of [(η^5^-C_5_Me_5_)(CO)_2_FeBCl_2_] with [Pd(PCy_3_)_2_] *via* the loss of one of the tri­cyclo­hexyl­phosphane ligands (Braunschweig *et al.*, 2005*a* ▶). In the same year, the synthesis of the related compound [(η^5^-C_5_Me_5_)(CO)Fe(μ-BBr)(μ-CO)PdBr(PCy_3_)], which was spectroscopically characterized, was reported without structural proof (Braunschweig *et al.*, 2005*b* ▶). One year later, Braunschweig *et al.* further reported the synthesis of [(η^5^-C_5_Me_5_)(OC)Fe(μ-CO)Pt(PCy_3_)(μ-Br)Pt(PCy_3_)Br(μ^3^-B)]. Spectroscopic investigations indicated that [(η^5^-C_5_Me_5_)(CO)Fe(μ-BBr)(μ-CO)PtBr(PCy_3_)] is likely initially formed, and subsequently reacts with a second equivalent of [Pt(PCy_3_)_2_] to give the final product. However, once again, no structural proof could be given (Braunschweig *et al.*, 2006 ▶).
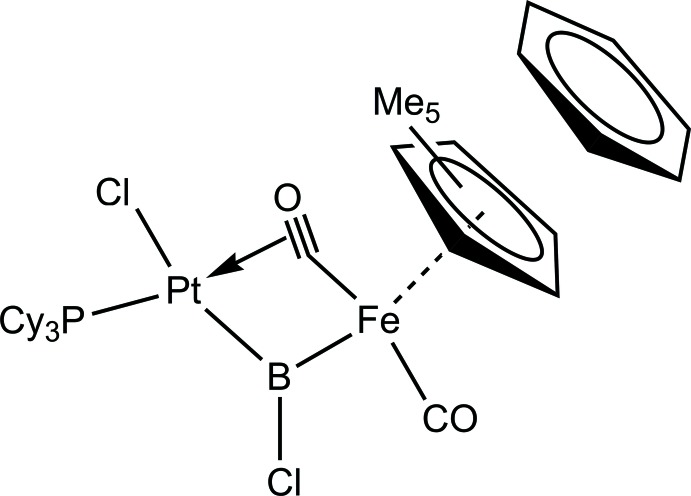


Herein we report the related heterodinuclear bridging chlorido­borylene complex [(η^5^-C_5_Me_5_)(CO)Fe{(μ-BCl)(μ-CO)}Pt(PCy_3_)Cl]·C_6_H_6_, (I), which forms upon the reaction of [Pt(PCy_3_)_2_] with [(η^5^-C_5_Me_5_)(CO)_2_FeBCl_2_] *via* oxidative addition of a B—Cl bond to the low-valent platinum, resulting in the loss of one phosphane ligand.

## Structural commentary   

**Figure 1:**
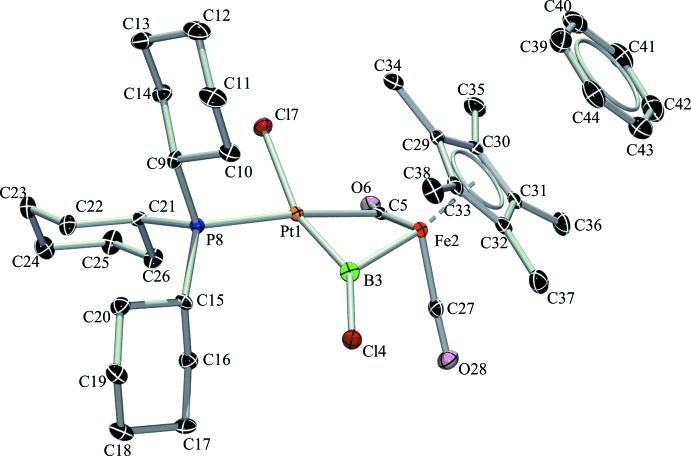
The mol­ecular structure of the title compound, showing the atom-numbering scheme and displacement ellipsoids for the non-H atoms at the 50% probability level. H atoms have been omitted for clarity.

The mol­ecular structure of compound (I) is shown in Fig. 1 ▶. As already reported for these type of reactions, the chloride ligand at the Pt atom adopts the *trans* position relative to the borylene unit due to its *trans* influence (Braunschweig *et al.*, 2010 ▶). The Fe—Pt distance of 2.6455 (5) Å is slightly longer than the sum of the covalent radii and is most likely influenced by the two bridging ligands between both metals. The bridging borylene ligand and the additional semi-bridging carbonyl ligand, together with the phosphane and chloride ligand, form a distorted square-planar structural motif at the Pt atom (Fig. 1 ▶). The Pt—B bond length [1.910 (4) Å] is shorter than the Fe—B bond length [2.009 (4) Å], indicating a stronger bonding inter­action. Compared to the similar parent compound [(η^5^-C_5_H_5_)(CO)_2_FeBCl_2_], (II), which has a Fe—B bond length of 1.942 (3) Å, there is an obvious lengthening of this bond in the target mol­ecule. In the structure of (I), the Fe atom is additionally bound to a (penta­meth­yl)cyclo­penta­dienyl ligand (η^5^-C_5_Me_5_) and one carbonyl ligand (CO), forming an overall piano-stool structure. The ^11^B NMR resonance in the spectrum of (I) is shifted downfield to 107.4 p.p.m. from the previous resonance at 95.3 p.p.m. in compound (II). The ^31^P NMR spectrum shows a peak at 56.55 p.p.m. with a coupling constant of ^1^ *J*_P–Pt_ = 4864 Hz, which is typical for a bridging square-planar platinum complex (Arnold *et al.*, 2012 ▶). Furthermore, the observed FT–IR signals are indicative of one semi-bridging carbonyl ligand at 1913 cm^−1^ and one terminal carbonyl ligand at 1978 cm^−1^.

## Supra­molecular features   

**Figure 2:**
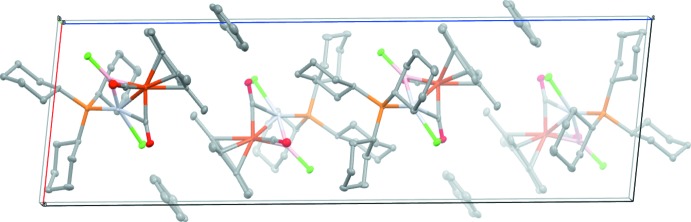
Packing plot of the title compound.

The orientation of the benzene solvent mol­ecule in the crystal structure of (I) with its staggered conformation with respect to the (penta­meth­yl)cyclo­penta­dienyl ligand and a centroid–centroid distance of 3.630 (2) Å (Fig. 1 ▶) raises the possibility of inter­molecular inter­actions, such as π–π stacking. However, as no further inter­actions are detected in the crystal structure (Fig. 2 ▶), it seems that the benzene mol­ecule occupies a free void in the asymmetric unit and mainly supports the crystallization process.

## Synthesis and crystallization   

[(η^5^-C_5_Me_5_)(CO)_2_Fe(BCl_2_)] (50.0 mg, 0.11 mmol) was dis­solved in 2 ml of benzene and bis­(tri­cyclo­hexyl­phos­phane)platinum (86.9 mg, 0.11 mmol) was added to the solution. After 5 h of stirring, the solvent was removed, by-products were extracted with two portions of 2 ml of hexane, and the bright-yellow residue was redissolved in 2 ml of benzene. Upon slow evaporation, yellow crystals suitable for X-ray diffraction were obtained at room temperature (yield: 72.4 mg, 0.09 mmol, 82%). Elemental analysis calculated (%): C 49.00, H 6.16; found (%): C 50.08, H 6.20. ^1^H NMR (C_6_D_6_, 400.1 MHz): δ 1.14–2.12 (30H, P*Cy_3_*), 1.66 (*s*, 15H, C_5_Me_5_), 2.92 (*m*, 3H, PC*H*). ^11^B{^1^H} NMR (C_6_D_6_, 128.4 MHz): δ 107.4. ^13^C{^1^H} NMR (C_6_D_6_, 100.6 MHz): δ 10.1 (C_5_ *Me*_5_), 27.0 (P*Cy_3_*), 27.8 (P*Cy_3_*), 30.6 (P*Cy_3_*), 34.4 (P*Cy_3_*), 98.1 (s, *C*_5_Me_5_), 167.6 (μ-CO), 205.9 (CO). ^31^P{^1^H} NMR (C_6_D_6_, 162.0 MHz): δ 56.55 (^1^ *J*_P–Pt_ = 4864 Hz). IR (toluene): 1978, 1913 cm^−1^.

## Refinement   

Crystal data, data collection and structure refinement details are summarized in Table 1 ▶. H atoms were placed at idealized positions and treated as riding atoms; C—H = 0.98 (CH_3_) or 1.00 Å (aliphatic). *U*_iso_(H) values were fixed at 1.5 (for primary H atoms) and 1.2 times (tertiary H atoms) *U*_eq_ of the parent C atoms.

## Supplementary Material

Crystal structure: contains datablock(s) I. DOI: 10.1107/S1600536814023381/wm5073sup1.cif

Structure factors: contains datablock(s) I. DOI: 10.1107/S1600536814023381/wm5073Isup2.hkl

CCDC reference: 1030674

Additional supporting information:  crystallographic information; 3D view; checkCIF report

## Figures and Tables

**Table 1:** Experimental details

Crystal data	
Chemical formula	[FePt(BCl)Cl(C_10_H_15_)(C_18_H_33_P)(CO)_2_]C_6_H_6_
*M*_r_	882.41
Crystal system, space group	Monoclinic, *P*2_1_/*c*
Temperature (K)	100
*a*, *b*, *c* ()	9.4578(2), 12.6902(3), 30.5130(9)
()	96.334(1)
*V* (^3^)	3639.86(16)
*Z*	4
Radiation type	Mo *K*
(mm^1^)	4.46
Crystal size (mm)	0.28 0.26 0.19

Data collection	
Diffractometer	Bruker X8 APEXII
Absorption correction	Multi-scan (*SADABS*; Bruker, 2009 ▶)
*T*_min_, *T*_max_	0.374, 0.431
No. of measured, independent and observed [*I* > 2(*I*)] reflections	57360, 9098, 6917
*R*_int_	0.077
(sin /)_max_ (^1^)	0.676

Refinement	
*R*[*F* ^2^ > 2(*F* ^2^)], *wR*(*F* ^2^), *S*	0.036, 0.059, 1.00
No. of reflections	9098
No. of parameters	402
H-atom treatment	H-atom parameters constrained
_max_, _min_ (e ^3^)	1.01, 0.78

Computer programs: *APEX2* and *SAINT-Plus* (Bruker, 2009 ▶), *SHELXS97* and *SHELXL97* (Sheldrick, 2008 ▶) and *SHELXLE* (Hbschle *et al.*, 2011 ▶).

## supplementary crystallographic information

### Crystal data

**Table:**

[FePt(BCl)Cl(C_10_H_15_)(C_18_H_33_P)(CO)_2_]·C_6_H_6_	*F*(000) = 1776
*M**_r_* = 882.41	*D*_x_ = 1.610 Mg m^−^^3^
Monoclinic, *P*2_1_/*c*	Mo *K*α radiation, λ = 0.71073 Å
Hall symbol: -P 2ybc	Cell parameters from 5551 reflections
*a* = 9.4578 (2) Å	θ = 2.6–22.4°
*b* = 12.6902 (3) Å	µ = 4.46 mm^−^^1^
*c* = 30.5130 (9) Å	*T* = 100 K
β = 96.334 (1)°	Block, yellow
*V* = 3639.86 (16) Å^3^	0.28 × 0.26 × 0.19 mm
*Z* = 4	

### Data collection

**Table:**

Bruker X8 APEXII diffractometer	9098 independent reflections
Radiation source: rotating anode	6917 reflections with *I* > 2σ(*I*)
Multi-layer mirror monochromator	*R*_int_ = 0.077
Detector resolution: 8.333 pixels mm^-1^	θ_max_ = 28.7°, θ_min_ = 1.3°
φ and ω scans	*h* = −12→12
Absorption correction: multi-scan (*SADABS*; Bruker, 2008)	*k* = −16→16
*T*_min_ = 0.374, *T*_max_ = 0.431	*l* = −41→39
57360 measured reflections	

### Refinement

**Table:**

Refinement on *F*^2^	Primary atom site location: structure-invariant direct methods
Least-squares matrix: full	Secondary atom site location: difference Fourier map
*R*[*F*^2^ > 2σ(*F*^2^)] = 0.036	Hydrogen site location: inferred from neighbouring sites
*wR*(*F*^2^) = 0.059	H-atom parameters constrained
*S* = 1.00	*w* = 1/[σ^2^(*F*_o_^2^) + (0.0177*P*)^2^] where *P* = (*F*_o_^2^ + 2*F*_c_^2^)/3
9098 reflections	(Δ/σ)_max_ = 0.001
402 parameters	Δρ_max_ = 1.01 e Å^−^^3^
0 restraints	Δρ_min_ = −0.78 e Å^−^^3^

### Special details

**Table:**

Experimental. The crystal was immersed in a film of perfluoropolyether oil, mounted on a glass fiber and transferred to stream of cold nitrogen.
Geometry. All e.s.d.'s (except the e.s.d. in the dihedral angle between two l.s. planes) are estimated using the full covariance matrix. The cell e.s.d.'s are taken into account individually in the estimation of e.s.d.'s in distances, angles and torsion angles; correlations between e.s.d.'s in cell parameters are only used when they are defined by crystal symmetry. An approximate (isotropic) treatment of cell e.s.d.'s is used for estimating e.s.d.'s involving l.s. planes.
Refinement. Refinement of *F*^2^ against ALL reflections. The weighted *R*-factor *wR* and goodness of fit *S* are based on *F*^2^, conventional *R*-factors *R* are based on *F*, with *F* set to zero for negative *F*^2^. The threshold expression of *F*^2^ > σ(*F*^2^) is used only for calculating *R*-factors(gt) *etc*. and is not relevant to the choice of reflections for refinement. *R*-factors based on *F*^2^ are statistically about twice as large as those based on *F*, and *R*- factors based on ALL data will be even larger.

### Fractional atomic coordinates and isotropic or equivalent isotropic displacement parameters (Å^2^)

**Table:**

	*x*	*y*	*z*	*U*_iso_*/*U*_eq_	
Pt1	0.526460 (14)	0.632771 (10)	0.387705 (4)	0.01107 (4)	
Fe2	0.64690 (5)	0.47736 (4)	0.347519 (14)	0.01174 (11)	
B3	0.6882 (4)	0.5475 (3)	0.40656 (12)	0.0134 (9)	
Cl4	0.82199 (9)	0.51932 (7)	0.45042 (3)	0.0170 (2)	
C5	0.4564 (4)	0.4860 (3)	0.34065 (10)	0.0136 (8)	
O6	0.3379 (3)	0.46715 (18)	0.32991 (7)	0.0181 (6)	
Cl7	0.32826 (9)	0.71629 (7)	0.34063 (3)	0.0182 (2)	
P8	0.52924 (9)	0.76039 (7)	0.43998 (3)	0.0109 (2)	
C9	0.6047 (3)	0.8854 (2)	0.42227 (10)	0.0118 (8)	
H9	0.6208	0.9316	0.4489	0.014*	
C10	0.7503 (4)	0.8649 (3)	0.40638 (11)	0.0176 (8)	
H10A	0.8123	0.8289	0.4301	0.021*	
H10B	0.7383	0.8172	0.3805	0.021*	
C11	0.8229 (4)	0.9664 (3)	0.39367 (11)	0.0219 (9)	
H11A	0.8470	1.0100	0.4203	0.026*	
H11B	0.9126	0.9487	0.3814	0.026*	
C12	0.7275 (4)	1.0286 (3)	0.35996 (11)	0.0248 (9)	
H12A	0.7140	0.9888	0.3319	0.030*	
H12B	0.7737	1.0965	0.3543	0.030*	
C13	0.5827 (4)	1.0497 (3)	0.37603 (11)	0.0213 (9)	
H13A	0.5215	1.0880	0.3529	0.026*	
H13B	0.5956	1.0950	0.4026	0.026*	
C14	0.5095 (4)	0.9472 (3)	0.38706 (11)	0.0162 (8)	
H14A	0.4903	0.9038	0.3601	0.019*	
H14B	0.4174	0.9634	0.3981	0.019*	
C15	0.6401 (4)	0.7299 (3)	0.49250 (10)	0.0119 (8)	
H15	0.7330	0.7041	0.4839	0.014*	
C16	0.6773 (4)	0.8230 (3)	0.52455 (10)	0.0160 (8)	
H16A	0.7249	0.8792	0.5091	0.019*	
H16B	0.5886	0.8527	0.5340	0.019*	
C17	0.7750 (4)	0.7875 (3)	0.56515 (10)	0.0184 (8)	
H17A	0.8672	0.7641	0.5560	0.022*	
H17B	0.7933	0.8476	0.5857	0.022*	
C18	0.7083 (4)	0.6977 (3)	0.58863 (11)	0.0193 (9)	
H18A	0.6189	0.7222	0.5994	0.023*	
H18B	0.7740	0.6751	0.6144	0.023*	
C19	0.6768 (4)	0.6048 (3)	0.55734 (11)	0.0191 (9)	
H19A	0.7669	0.5774	0.5481	0.023*	
H19B	0.6312	0.5476	0.5728	0.023*	
C20	0.5788 (4)	0.6385 (3)	0.51681 (10)	0.0167 (8)	
H20A	0.4856	0.6596	0.5260	0.020*	
H20B	0.5626	0.5777	0.4965	0.020*	
C21	0.3461 (3)	0.7936 (3)	0.44995 (10)	0.0123 (8)	
H21	0.3024	0.8244	0.4214	0.015*	
C22	0.3291 (4)	0.8798 (3)	0.48436 (11)	0.0156 (8)	
H22A	0.3639	0.8534	0.5141	0.019*	
H22B	0.3868	0.9421	0.4782	0.019*	
C23	0.1729 (4)	0.9117 (3)	0.48306 (11)	0.0188 (8)	
H23A	0.1629	0.9665	0.5056	0.023*	
H23B	0.1399	0.9419	0.4538	0.023*	
C24	0.0816 (4)	0.8174 (3)	0.49171 (11)	0.0215 (9)	
H24A	−0.0193	0.8392	0.4900	0.026*	
H24B	0.1103	0.7901	0.5218	0.026*	
C25	0.0977 (4)	0.7308 (3)	0.45814 (12)	0.0220 (9)	
H25A	0.0407	0.6689	0.4652	0.026*	
H25B	0.0601	0.7561	0.4284	0.026*	
C26	0.2519 (4)	0.6978 (3)	0.45771 (12)	0.0173 (8)	
H26A	0.2589	0.6452	0.4341	0.021*	
H26B	0.2863	0.6644	0.4862	0.021*	
C27	0.6543 (4)	0.3662 (3)	0.38169 (10)	0.0148 (8)	
O28	0.6684 (3)	0.29309 (19)	0.40407 (8)	0.0213 (6)	
C29	0.7129 (4)	0.5825 (3)	0.29891 (10)	0.0143 (8)	
C30	0.6452 (4)	0.4909 (3)	0.27809 (10)	0.0136 (8)	
C31	0.7216 (4)	0.4009 (3)	0.29399 (10)	0.0134 (8)	
C32	0.8390 (3)	0.4352 (3)	0.32478 (10)	0.0139 (8)	
C33	0.8337 (4)	0.5478 (3)	0.32728 (10)	0.0149 (8)	
C34	0.6721 (4)	0.6938 (3)	0.28770 (11)	0.0213 (9)	
H34A	0.6897	0.7087	0.2573	0.032*	
H34B	0.7290	0.7417	0.3078	0.032*	
H34C	0.5710	0.7040	0.2907	0.032*	
C35	0.5207 (4)	0.4923 (3)	0.24333 (11)	0.0226 (9)	
H35A	0.5546	0.4933	0.2141	0.034*	
H35B	0.4631	0.5553	0.2469	0.034*	
H35C	0.4626	0.4292	0.2461	0.034*	
C36	0.6898 (4)	0.2893 (3)	0.27928 (11)	0.0233 (9)	
H36A	0.7157	0.2796	0.2493	0.035*	
H36B	0.5881	0.2751	0.2796	0.035*	
H36C	0.7450	0.2405	0.2993	0.035*	
C37	0.9540 (4)	0.3681 (3)	0.34783 (11)	0.0234 (9)	
H37A	1.0388	0.3721	0.3321	0.035*	
H37B	0.9213	0.2949	0.3484	0.035*	
H37C	0.9773	0.3934	0.3781	0.035*	
C38	0.9461 (4)	0.6162 (3)	0.35157 (11)	0.0230 (9)	
H38A	1.0189	0.6330	0.3322	0.035*	
H38B	0.9901	0.5787	0.3777	0.035*	
H38C	0.9027	0.6816	0.3607	0.035*	
C39	0.9832 (4)	0.6166 (3)	0.22311 (12)	0.0269 (10)	
H39	0.9887	0.6905	0.2281	0.032*	
C40	0.8760 (4)	0.5756 (3)	0.19333 (12)	0.0272 (10)	
H40	0.8083	0.6213	0.1778	0.033*	
C41	0.8680 (4)	0.4681 (3)	0.18633 (12)	0.0287 (10)	
H41	0.7941	0.4397	0.1662	0.034*	
C42	0.9662 (4)	0.4022 (3)	0.20839 (12)	0.0305 (10)	
H42	0.9606	0.3283	0.2035	0.037*	
C43	1.0738 (4)	0.4437 (3)	0.23779 (12)	0.0258 (9)	
H43	1.1425	0.3979	0.2528	0.031*	
C44	1.0823 (4)	0.5503 (3)	0.24558 (11)	0.0232 (9)	
H44	1.1554	0.5781	0.2662	0.028*	

### Atomic displacement parameters (Å^2^)

**Table:**

	*U*^11^	*U*^22^	*U*^33^	*U*^12^	*U*^13^	*U*^23^
Pt1	0.01238 (8)	0.01078 (8)	0.00994 (6)	0.00060 (6)	0.00079 (5)	−0.00084 (6)
Fe2	0.0135 (3)	0.0119 (3)	0.0099 (2)	0.0007 (2)	0.0015 (2)	−0.0012 (2)
B3	0.010 (2)	0.013 (2)	0.018 (2)	−0.0073 (18)	0.0023 (17)	−0.0013 (17)
Cl4	0.0163 (5)	0.0193 (5)	0.0146 (4)	0.0019 (4)	−0.0027 (4)	−0.0020 (4)
C5	0.021 (2)	0.012 (2)	0.0075 (16)	0.0010 (17)	0.0011 (15)	0.0000 (14)
O6	0.0154 (14)	0.0202 (15)	0.0178 (13)	−0.0010 (12)	−0.0024 (11)	−0.0005 (11)
Cl7	0.0188 (5)	0.0175 (5)	0.0172 (4)	0.0041 (4)	−0.0033 (4)	−0.0004 (4)
P8	0.0123 (5)	0.0104 (5)	0.0100 (4)	−0.0003 (4)	0.0012 (4)	−0.0006 (4)
C9	0.0116 (18)	0.0083 (19)	0.0153 (17)	−0.0028 (15)	0.0010 (14)	−0.0024 (14)
C10	0.018 (2)	0.019 (2)	0.0158 (17)	−0.0013 (17)	0.0034 (15)	0.0021 (16)
C11	0.021 (2)	0.025 (2)	0.021 (2)	−0.0107 (18)	0.0060 (17)	0.0051 (17)
C12	0.035 (3)	0.020 (2)	0.020 (2)	−0.0096 (19)	0.0058 (18)	0.0049 (17)
C13	0.029 (2)	0.016 (2)	0.0180 (19)	0.0008 (18)	0.0015 (17)	0.0045 (16)
C14	0.018 (2)	0.0130 (19)	0.0170 (18)	−0.0020 (16)	−0.0001 (16)	0.0001 (16)
C15	0.0131 (19)	0.0145 (19)	0.0074 (16)	−0.0026 (15)	−0.0013 (14)	−0.0016 (14)
C16	0.017 (2)	0.0138 (19)	0.0165 (18)	−0.0002 (16)	0.0002 (15)	0.0003 (15)
C17	0.018 (2)	0.024 (2)	0.0142 (18)	−0.0004 (17)	0.0030 (15)	0.0003 (16)
C18	0.015 (2)	0.030 (2)	0.0129 (18)	−0.0002 (18)	0.0012 (15)	0.0048 (17)
C19	0.025 (2)	0.016 (2)	0.0170 (18)	−0.0004 (17)	0.0053 (16)	0.0075 (15)
C20	0.019 (2)	0.016 (2)	0.0153 (17)	−0.0006 (17)	0.0006 (15)	0.0000 (16)
C21	0.0140 (19)	0.0097 (19)	0.0127 (17)	0.0011 (15)	−0.0002 (14)	0.0009 (14)
C22	0.016 (2)	0.011 (2)	0.0202 (18)	−0.0048 (16)	0.0039 (15)	−0.0035 (15)
C23	0.018 (2)	0.018 (2)	0.0207 (19)	0.0006 (17)	0.0050 (16)	−0.0052 (16)
C24	0.012 (2)	0.030 (2)	0.022 (2)	0.0007 (18)	0.0040 (16)	−0.0025 (17)
C25	0.014 (2)	0.019 (2)	0.033 (2)	−0.0066 (17)	0.0037 (17)	−0.0046 (18)
C26	0.015 (2)	0.013 (2)	0.025 (2)	−0.0057 (16)	0.0057 (16)	0.0001 (16)
C27	0.018 (2)	0.013 (2)	0.0124 (17)	−0.0010 (17)	0.0005 (15)	−0.0055 (16)
O28	0.0244 (15)	0.0160 (14)	0.0234 (14)	0.0010 (12)	0.0019 (12)	0.0018 (12)
C29	0.016 (2)	0.016 (2)	0.0129 (17)	0.0027 (16)	0.0078 (15)	0.0020 (15)
C30	0.016 (2)	0.016 (2)	0.0101 (16)	−0.0003 (16)	0.0061 (14)	0.0023 (15)
C31	0.0138 (19)	0.019 (2)	0.0082 (16)	0.0017 (16)	0.0026 (14)	−0.0024 (15)
C32	0.0104 (19)	0.021 (2)	0.0100 (17)	0.0004 (16)	0.0022 (14)	−0.0003 (15)
C33	0.016 (2)	0.018 (2)	0.0121 (17)	−0.0005 (16)	0.0069 (15)	−0.0007 (15)
C34	0.032 (2)	0.018 (2)	0.0151 (19)	0.0010 (18)	0.0080 (17)	0.0029 (16)
C35	0.022 (2)	0.031 (2)	0.0143 (18)	0.0019 (19)	−0.0019 (16)	0.0002 (17)
C36	0.031 (2)	0.021 (2)	0.0183 (19)	−0.0013 (19)	0.0054 (17)	−0.0090 (17)
C37	0.018 (2)	0.033 (2)	0.0186 (19)	0.0073 (19)	0.0025 (16)	−0.0026 (18)
C38	0.021 (2)	0.029 (2)	0.0198 (19)	−0.0026 (18)	0.0046 (16)	−0.0056 (17)
C39	0.033 (2)	0.020 (2)	0.030 (2)	0.000 (2)	0.0122 (19)	−0.0004 (18)
C40	0.021 (2)	0.040 (3)	0.022 (2)	0.004 (2)	0.0079 (17)	0.0073 (19)
C41	0.028 (2)	0.040 (3)	0.019 (2)	−0.010 (2)	0.0045 (18)	−0.0072 (19)
C42	0.042 (3)	0.024 (2)	0.029 (2)	−0.006 (2)	0.018 (2)	−0.0043 (19)
C43	0.028 (2)	0.028 (2)	0.023 (2)	0.001 (2)	0.0075 (18)	0.0034 (18)
C44	0.018 (2)	0.033 (3)	0.0193 (19)	−0.0090 (19)	0.0071 (16)	−0.0069 (18)

### Geometric parameters (Å, º)

**Table:**

Pt1—B3	1.910 (4)	C21—H21	1.0000
Pt1—P8	2.2712 (9)	C22—C23	1.528 (5)
Pt1—C5	2.400 (3)	C22—H22A	0.9900
Pt1—Cl7	2.4711 (8)	C22—H22B	0.9900
Pt1—Fe2	2.6455 (5)	C23—C24	1.516 (5)
Fe2—C27	1.751 (4)	C23—H23A	0.9900
Fe2—C5	1.794 (4)	C23—H23B	0.9900
Fe2—B3	2.009 (4)	C24—C25	1.521 (5)
Fe2—C32	2.085 (3)	C24—H24A	0.9900
Fe2—C31	2.089 (3)	C24—H24B	0.9900
Fe2—C30	2.124 (3)	C25—C26	1.519 (5)
Fe2—C33	2.132 (3)	C25—H25A	0.9900
Fe2—C29	2.138 (3)	C25—H25B	0.9900
B3—Cl4	1.774 (4)	C26—H26A	0.9900
C5—O6	1.158 (4)	C26—H26B	0.9900
P8—C21	1.841 (3)	C27—O28	1.151 (4)
P8—C9	1.845 (3)	C29—C33	1.425 (5)
P8—C15	1.856 (3)	C29—C30	1.441 (5)
C9—C10	1.533 (5)	C29—C34	1.494 (5)
C9—C14	1.538 (4)	C30—C31	1.409 (5)
C9—H9	1.0000	C30—C35	1.495 (4)
C10—C11	1.529 (5)	C31—C32	1.440 (4)
C10—H10A	0.9900	C31—C36	1.505 (5)
C10—H10B	0.9900	C32—C33	1.432 (5)
C11—C12	1.514 (5)	C32—C37	1.495 (5)
C11—H11A	0.9900	C33—C38	1.503 (5)
C11—H11B	0.9900	C34—H34A	0.9800
C12—C13	1.528 (5)	C34—H34B	0.9800
C12—H12A	0.9900	C34—H34C	0.9800
C12—H12B	0.9900	C35—H35A	0.9800
C13—C14	1.529 (5)	C35—H35B	0.9800
C13—H13A	0.9900	C35—H35C	0.9800
C13—H13B	0.9900	C36—H36A	0.9800
C14—H14A	0.9900	C36—H36B	0.9800
C14—H14B	0.9900	C36—H36C	0.9800
C15—C20	1.526 (5)	C37—H37A	0.9800
C15—C16	1.550 (4)	C37—H37B	0.9800
C15—H15	1.0000	C37—H37C	0.9800
C16—C17	1.530 (4)	C38—H38A	0.9800
C16—H16A	0.9900	C38—H38B	0.9800
C16—H16B	0.9900	C38—H38C	0.9800
C17—C18	1.519 (5)	C39—C44	1.384 (5)
C17—H17A	0.9900	C39—C40	1.386 (5)
C17—H17B	0.9900	C39—H39	0.9500
C18—C19	1.526 (5)	C40—C41	1.383 (5)
C18—H18A	0.9900	C40—H40	0.9500
C18—H18B	0.9900	C41—C42	1.370 (5)
C19—C20	1.522 (4)	C41—H41	0.9500
C19—H19A	0.9900	C42—C43	1.384 (5)
C19—H19B	0.9900	C42—H42	0.9500
C20—H20A	0.9900	C43—C44	1.374 (5)
C20—H20B	0.9900	C43—H43	0.9500
C21—C22	1.537 (4)	C44—H44	0.9500
C21—C26	1.541 (5)		
			
B3—Pt1—P8	104.22 (12)	H19A—C19—H19B	108.1
B3—Pt1—C5	84.23 (14)	C19—C20—C15	112.2 (3)
P8—Pt1—C5	162.67 (9)	C19—C20—H20A	109.2
B3—Pt1—Cl7	161.64 (12)	C15—C20—H20A	109.2
P8—Pt1—Cl7	92.96 (3)	C19—C20—H20B	109.2
C5—Pt1—Cl7	81.00 (8)	C15—C20—H20B	109.2
B3—Pt1—Fe2	49.13 (11)	H20A—C20—H20B	107.9
P8—Pt1—Fe2	152.23 (3)	C22—C21—C26	110.6 (3)
C5—Pt1—Fe2	41.29 (9)	C22—C21—P8	116.5 (2)
Cl7—Pt1—Fe2	112.84 (2)	C26—C21—P8	114.5 (2)
C27—Fe2—C5	95.32 (16)	C22—C21—H21	104.6
C27—Fe2—B3	80.32 (15)	C26—C21—H21	104.6
C5—Fe2—B3	99.86 (15)	P8—C21—H21	104.6
C27—Fe2—C32	90.69 (15)	C23—C22—C21	110.1 (3)
C5—Fe2—C32	151.34 (13)	C23—C22—H22A	109.6
B3—Fe2—C32	108.78 (14)	C21—C22—H22A	109.6
C27—Fe2—C31	95.53 (14)	C23—C22—H22B	109.6
C5—Fe2—C31	111.03 (14)	C21—C22—H22B	109.6
B3—Fe2—C31	149.10 (15)	H22A—C22—H22B	108.2
C32—Fe2—C31	40.37 (12)	C24—C23—C22	110.8 (3)
C27—Fe2—C30	130.89 (14)	C24—C23—H23A	109.5
C5—Fe2—C30	88.93 (14)	C22—C23—H23A	109.5
B3—Fe2—C30	146.94 (15)	C24—C23—H23B	109.5
C32—Fe2—C30	66.41 (12)	C22—C23—H23B	109.5
C31—Fe2—C30	39.07 (12)	H23A—C23—H23B	108.1
C27—Fe2—C33	122.02 (15)	C23—C24—C25	110.6 (3)
C5—Fe2—C33	142.60 (14)	C23—C24—H24A	109.5
B3—Fe2—C33	89.34 (14)	C25—C24—H24A	109.5
C32—Fe2—C33	39.70 (13)	C23—C24—H24B	109.5
C31—Fe2—C33	66.78 (13)	C25—C24—H24B	109.5
C30—Fe2—C33	65.95 (13)	H24A—C24—H24B	108.1
C27—Fe2—C29	156.92 (15)	C26—C25—C24	111.9 (3)
C5—Fe2—C29	104.29 (14)	C26—C25—H25A	109.2
B3—Fe2—C29	107.62 (15)	C24—C25—H25A	109.2
C32—Fe2—C29	66.28 (13)	C26—C25—H25B	109.2
C31—Fe2—C29	66.29 (13)	C24—C25—H25B	109.2
C30—Fe2—C29	39.51 (12)	H25A—C25—H25B	107.9
C33—Fe2—C29	39.00 (12)	C25—C26—C21	110.8 (3)
C27—Fe2—Pt1	108.40 (11)	C25—C26—H26A	109.5
C5—Fe2—Pt1	61.99 (10)	C21—C26—H26A	109.5
B3—Fe2—Pt1	45.98 (12)	C25—C26—H26B	109.5
C32—Fe2—Pt1	141.37 (10)	C21—C26—H26B	109.5
C31—Fe2—Pt1	155.35 (9)	H26A—C26—H26B	108.1
C30—Fe2—Pt1	116.48 (9)	O28—C27—Fe2	175.7 (3)
C33—Fe2—Pt1	103.57 (9)	C33—C29—C30	107.8 (3)
C29—Fe2—Pt1	91.71 (9)	C33—C29—C34	126.9 (3)
Cl4—B3—Pt1	145.3 (2)	C30—C29—C34	124.8 (3)
Cl4—B3—Fe2	129.7 (2)	C33—C29—Fe2	70.24 (19)
Pt1—B3—Fe2	84.89 (15)	C30—C29—Fe2	69.70 (18)
O6—C5—Fe2	161.6 (3)	C34—C29—Fe2	131.4 (2)
O6—C5—Pt1	121.6 (3)	C31—C30—C29	108.4 (3)
Fe2—C5—Pt1	76.72 (12)	C31—C30—C35	126.0 (3)
C21—P8—C9	104.64 (15)	C29—C30—C35	125.5 (3)
C21—P8—C15	110.93 (15)	C31—C30—Fe2	69.13 (18)
C9—P8—C15	103.45 (15)	C29—C30—Fe2	70.79 (18)
C21—P8—Pt1	109.95 (11)	C35—C30—Fe2	128.8 (2)
C9—P8—Pt1	112.48 (11)	C30—C31—C32	108.0 (3)
C15—P8—Pt1	114.79 (11)	C30—C31—C36	125.6 (3)
C10—C9—C14	110.1 (3)	C32—C31—C36	126.4 (3)
C10—C9—P8	109.7 (2)	C30—C31—Fe2	71.80 (19)
C14—C9—P8	115.3 (2)	C32—C31—Fe2	69.65 (18)
C10—C9—H9	107.1	C36—C31—Fe2	126.6 (2)
C14—C9—H9	107.1	C33—C32—C31	107.9 (3)
P8—C9—H9	107.1	C33—C32—C37	124.8 (3)
C11—C10—C9	112.4 (3)	C31—C32—C37	127.1 (3)
C11—C10—H10A	109.1	C33—C32—Fe2	71.91 (19)
C9—C10—H10A	109.1	C31—C32—Fe2	69.97 (18)
C11—C10—H10B	109.1	C37—C32—Fe2	127.3 (2)
C9—C10—H10B	109.1	C29—C33—C32	107.8 (3)
H10A—C10—H10B	107.9	C29—C33—C38	126.7 (3)
C12—C11—C10	111.2 (3)	C32—C33—C38	125.2 (3)
C12—C11—H11A	109.4	C29—C33—Fe2	70.76 (19)
C10—C11—H11A	109.4	C32—C33—Fe2	68.39 (19)
C12—C11—H11B	109.4	C38—C33—Fe2	131.5 (2)
C10—C11—H11B	109.4	C29—C34—H34A	109.5
H11A—C11—H11B	108.0	C29—C34—H34B	109.5
C11—C12—C13	111.2 (3)	H34A—C34—H34B	109.5
C11—C12—H12A	109.4	C29—C34—H34C	109.5
C13—C12—H12A	109.4	H34A—C34—H34C	109.5
C11—C12—H12B	109.4	H34B—C34—H34C	109.5
C13—C12—H12B	109.4	C30—C35—H35A	109.5
H12A—C12—H12B	108.0	C30—C35—H35B	109.5
C12—C13—C14	111.4 (3)	H35A—C35—H35B	109.5
C12—C13—H13A	109.3	C30—C35—H35C	109.5
C14—C13—H13A	109.3	H35A—C35—H35C	109.5
C12—C13—H13B	109.3	H35B—C35—H35C	109.5
C14—C13—H13B	109.3	C31—C36—H36A	109.5
H13A—C13—H13B	108.0	C31—C36—H36B	109.5
C13—C14—C9	110.1 (3)	H36A—C36—H36B	109.5
C13—C14—H14A	109.6	C31—C36—H36C	109.5
C9—C14—H14A	109.6	H36A—C36—H36C	109.5
C13—C14—H14B	109.6	H36B—C36—H36C	109.5
C9—C14—H14B	109.6	C32—C37—H37A	109.5
H14A—C14—H14B	108.2	C32—C37—H37B	109.5
C20—C15—C16	110.2 (3)	H37A—C37—H37B	109.5
C20—C15—P8	111.7 (2)	C32—C37—H37C	109.5
C16—C15—P8	116.9 (2)	H37A—C37—H37C	109.5
C20—C15—H15	105.7	H37B—C37—H37C	109.5
C16—C15—H15	105.7	C33—C38—H38A	109.5
P8—C15—H15	105.7	C33—C38—H38B	109.5
C17—C16—C15	111.2 (3)	H38A—C38—H38B	109.5
C17—C16—H16A	109.4	C33—C38—H38C	109.5
C15—C16—H16A	109.4	H38A—C38—H38C	109.5
C17—C16—H16B	109.4	H38B—C38—H38C	109.5
C15—C16—H16B	109.4	C44—C39—C40	120.2 (4)
H16A—C16—H16B	108.0	C44—C39—H39	119.9
C18—C17—C16	111.0 (3)	C40—C39—H39	119.9
C18—C17—H17A	109.4	C41—C40—C39	119.7 (4)
C16—C17—H17A	109.4	C41—C40—H40	120.2
C18—C17—H17B	109.4	C39—C40—H40	120.2
C16—C17—H17B	109.4	C42—C41—C40	120.2 (4)
H17A—C17—H17B	108.0	C42—C41—H41	119.9
C17—C18—C19	110.3 (3)	C40—C41—H41	119.9
C17—C18—H18A	109.6	C41—C42—C43	119.8 (4)
C19—C18—H18A	109.6	C41—C42—H42	120.1
C17—C18—H18B	109.6	C43—C42—H42	120.1
C19—C18—H18B	109.6	C44—C43—C42	120.7 (4)
H18A—C18—H18B	108.1	C44—C43—H43	119.6
C20—C19—C18	110.5 (3)	C42—C43—H43	119.6
C20—C19—H19A	109.5	C43—C44—C39	119.3 (4)
C18—C19—H19A	109.5	C43—C44—H44	120.3
C20—C19—H19B	109.5	C39—C44—H44	120.3
C18—C19—H19B	109.5		
			
B3—Pt1—Fe2—C27	55.40 (19)	C27—Fe2—C29—C34	−163.7 (3)
P8—Pt1—Fe2—C27	74.29 (13)	C5—Fe2—C29—C34	48.9 (3)
C5—Pt1—Fe2—C27	−86.21 (16)	B3—Fe2—C29—C34	−56.6 (3)
Cl7—Pt1—Fe2—C27	−128.75 (12)	C32—Fe2—C29—C34	−159.8 (4)
B3—Pt1—Fe2—C5	141.60 (19)	C31—Fe2—C29—C34	155.9 (4)
P8—Pt1—Fe2—C5	160.50 (12)	C30—Fe2—C29—C34	119.1 (4)
Cl7—Pt1—Fe2—C5	−42.54 (12)	C33—Fe2—C29—C34	−122.3 (4)
P8—Pt1—Fe2—B3	18.89 (16)	Pt1—Fe2—C29—C34	−12.6 (3)
C5—Pt1—Fe2—B3	−141.60 (19)	C33—C29—C30—C31	1.1 (4)
Cl7—Pt1—Fe2—B3	175.86 (15)	C34—C29—C30—C31	173.9 (3)
B3—Pt1—Fe2—C32	−60.5 (2)	Fe2—C29—C30—C31	−59.1 (2)
P8—Pt1—Fe2—C32	−41.60 (16)	C33—C29—C30—C35	−175.3 (3)
C5—Pt1—Fe2—C32	157.90 (18)	C34—C29—C30—C35	−2.4 (5)
Cl7—Pt1—Fe2—C32	115.36 (14)	Fe2—C29—C30—C35	124.6 (3)
B3—Pt1—Fe2—C31	−139.0 (3)	C33—C29—C30—Fe2	60.2 (2)
P8—Pt1—Fe2—C31	−120.1 (2)	C34—C29—C30—Fe2	−127.0 (3)
C5—Pt1—Fe2—C31	79.4 (3)	C27—Fe2—C30—C31	−30.2 (3)
Cl7—Pt1—Fe2—C31	36.8 (2)	C5—Fe2—C30—C31	−126.3 (2)
B3—Pt1—Fe2—C30	−145.16 (18)	B3—Fe2—C30—C31	127.1 (3)
P8—Pt1—Fe2—C30	−126.27 (11)	C32—Fe2—C30—C31	38.6 (2)
C5—Pt1—Fe2—C30	73.24 (16)	C33—Fe2—C30—C31	82.2 (2)
Cl7—Pt1—Fe2—C30	30.69 (11)	C29—Fe2—C30—C31	119.4 (3)
B3—Pt1—Fe2—C33	−75.53 (18)	Pt1—Fe2—C30—C31	175.94 (16)
P8—Pt1—Fe2—C33	−56.64 (11)	C27—Fe2—C30—C29	−149.6 (2)
C5—Pt1—Fe2—C33	142.87 (15)	C5—Fe2—C30—C29	114.3 (2)
Cl7—Pt1—Fe2—C33	100.33 (9)	B3—Fe2—C30—C29	7.7 (4)
B3—Pt1—Fe2—C29	−113.10 (18)	C32—Fe2—C30—C29	−80.8 (2)
P8—Pt1—Fe2—C29	−94.20 (11)	C31—Fe2—C30—C29	−119.4 (3)
C5—Pt1—Fe2—C29	105.30 (15)	C33—Fe2—C30—C29	−37.20 (19)
Cl7—Pt1—Fe2—C29	62.76 (10)	Pt1—Fe2—C30—C29	56.5 (2)
P8—Pt1—B3—Cl4	11.6 (4)	C27—Fe2—C30—C35	89.8 (4)
C5—Pt1—B3—Cl4	−153.0 (4)	C5—Fe2—C30—C35	−6.3 (3)
Cl7—Pt1—B3—Cl4	170.45 (11)	B3—Fe2—C30—C35	−112.9 (4)
Fe2—Pt1—B3—Cl4	−177.3 (5)	C32—Fe2—C30—C35	158.7 (4)
P8—Pt1—B3—Fe2	−171.05 (8)	C31—Fe2—C30—C35	120.0 (4)
C5—Pt1—B3—Fe2	24.33 (12)	C33—Fe2—C30—C35	−157.7 (4)
Cl7—Pt1—B3—Fe2	−12.2 (4)	C29—Fe2—C30—C35	−120.5 (4)
C27—Fe2—B3—Cl4	50.4 (3)	Pt1—Fe2—C30—C35	−64.0 (3)
C5—Fe2—B3—Cl4	144.2 (3)	C29—C30—C31—C32	−0.4 (4)
C32—Fe2—B3—Cl4	−37.0 (3)	C35—C30—C31—C32	175.9 (3)
C31—Fe2—B3—Cl4	−34.1 (5)	Fe2—C30—C31—C32	−60.6 (2)
C30—Fe2—B3—Cl4	−112.3 (3)	C29—C30—C31—C36	−177.4 (3)
C33—Fe2—B3—Cl4	−72.2 (3)	C35—C30—C31—C36	−1.1 (5)
C29—Fe2—B3—Cl4	−107.2 (3)	Fe2—C30—C31—C36	122.5 (3)
Pt1—Fe2—B3—Cl4	178.0 (4)	C29—C30—C31—Fe2	60.1 (2)
C27—Fe2—B3—Pt1	−127.60 (16)	C35—C30—C31—Fe2	−123.6 (3)
C5—Fe2—B3—Pt1	−33.82 (16)	C27—Fe2—C31—C30	157.5 (2)
C32—Fe2—B3—Pt1	144.98 (13)	C5—Fe2—C31—C30	59.7 (2)
C31—Fe2—B3—Pt1	147.8 (2)	B3—Fe2—C31—C30	−122.1 (3)
C30—Fe2—B3—Pt1	69.6 (3)	C32—Fe2—C31—C30	−117.9 (3)
C33—Fe2—B3—Pt1	109.73 (14)	C33—Fe2—C31—C30	−79.9 (2)
C29—Fe2—B3—Pt1	74.73 (15)	C29—Fe2—C31—C30	−37.25 (19)
C27—Fe2—C5—O6	−69.1 (9)	Pt1—Fe2—C31—C30	−8.7 (4)
B3—Fe2—C5—O6	−150.2 (9)	C27—Fe2—C31—C32	−84.5 (2)
C32—Fe2—C5—O6	32.2 (11)	C5—Fe2—C31—C32	177.6 (2)
C31—Fe2—C5—O6	28.9 (9)	B3—Fe2—C31—C32	−4.2 (4)
C30—Fe2—C5—O6	61.8 (9)	C30—Fe2—C31—C32	117.9 (3)
C33—Fe2—C5—O6	107.8 (9)	C33—Fe2—C31—C32	38.01 (19)
C29—Fe2—C5—O6	98.6 (9)	C29—Fe2—C31—C32	80.7 (2)
Pt1—Fe2—C5—O6	−177.2 (10)	Pt1—Fe2—C31—C32	109.2 (2)
C27—Fe2—C5—Pt1	108.03 (12)	C27—Fe2—C31—C36	36.3 (3)
B3—Fe2—C5—Pt1	26.96 (14)	C5—Fe2—C31—C36	−61.6 (3)
C32—Fe2—C5—Pt1	−150.7 (3)	B3—Fe2—C31—C36	116.7 (4)
C31—Fe2—C5—Pt1	−153.95 (11)	C32—Fe2—C31—C36	120.8 (4)
C30—Fe2—C5—Pt1	−120.99 (11)	C30—Fe2—C31—C36	−121.2 (4)
C33—Fe2—C5—Pt1	−75.1 (2)	C33—Fe2—C31—C36	158.8 (3)
C29—Fe2—C5—Pt1	−84.23 (11)	C29—Fe2—C31—C36	−158.5 (3)
B3—Pt1—C5—O6	150.8 (3)	Pt1—Fe2—C31—C36	−130.0 (3)
P8—Pt1—C5—O6	30.4 (5)	C30—C31—C32—C33	−0.3 (4)
Cl7—Pt1—C5—O6	−40.2 (3)	C36—C31—C32—C33	176.6 (3)
Fe2—Pt1—C5—O6	178.9 (3)	Fe2—C31—C32—C33	−62.3 (2)
B3—Pt1—C5—Fe2	−28.17 (14)	C30—C31—C32—C37	−175.9 (3)
P8—Pt1—C5—Fe2	−148.5 (2)	C36—C31—C32—C37	1.0 (5)
Cl7—Pt1—C5—Fe2	140.88 (10)	Fe2—C31—C32—C37	122.2 (3)
B3—Pt1—P8—C21	−143.61 (16)	C30—C31—C32—Fe2	61.9 (2)
C5—Pt1—P8—C21	−26.0 (3)	C36—C31—C32—Fe2	−121.1 (3)
Cl7—Pt1—P8—C21	42.93 (11)	C27—Fe2—C32—C33	−144.6 (2)
Fe2—Pt1—P8—C21	−158.24 (11)	C5—Fe2—C32—C33	112.9 (3)
B3—Pt1—P8—C9	100.22 (16)	B3—Fe2—C32—C33	−64.6 (2)
C5—Pt1—P8—C9	−142.1 (3)	C31—Fe2—C32—C33	117.6 (3)
Cl7—Pt1—P8—C9	−73.25 (12)	C30—Fe2—C32—C33	80.2 (2)
Fe2—Pt1—P8—C9	85.58 (13)	C29—Fe2—C32—C33	36.90 (18)
B3—Pt1—P8—C15	−17.71 (17)	Pt1—Fe2—C32—C33	−23.3 (3)
C5—Pt1—P8—C15	99.9 (3)	C27—Fe2—C32—C31	97.7 (2)
Cl7—Pt1—P8—C15	168.82 (12)	C5—Fe2—C32—C31	−4.7 (4)
Fe2—Pt1—P8—C15	−32.35 (14)	B3—Fe2—C32—C31	177.7 (2)
C21—P8—C9—C10	−171.7 (2)	C30—Fe2—C32—C31	−37.42 (19)
C15—P8—C9—C10	72.0 (2)	C33—Fe2—C32—C31	−117.6 (3)
Pt1—P8—C9—C10	−52.4 (2)	C29—Fe2—C32—C31	−80.7 (2)
C21—P8—C9—C14	−46.8 (3)	Pt1—Fe2—C32—C31	−140.88 (17)
C15—P8—C9—C14	−163.1 (2)	C27—Fe2—C32—C37	−24.2 (3)
Pt1—P8—C9—C14	72.5 (3)	C5—Fe2—C32—C37	−126.7 (3)
C14—C9—C10—C11	55.4 (4)	B3—Fe2—C32—C37	55.8 (3)
P8—C9—C10—C11	−176.7 (2)	C31—Fe2—C32—C37	−122.0 (4)
C9—C10—C11—C12	−54.5 (4)	C30—Fe2—C32—C37	−159.4 (3)
C10—C11—C12—C13	54.3 (4)	C33—Fe2—C32—C37	120.4 (4)
C11—C12—C13—C14	−56.8 (4)	C29—Fe2—C32—C37	157.3 (3)
C12—C13—C14—C9	57.7 (4)	Pt1—Fe2—C32—C37	97.1 (3)
C10—C9—C14—C13	−56.4 (4)	C30—C29—C33—C32	−1.3 (4)
P8—C9—C14—C13	178.9 (2)	C34—C29—C33—C32	−174.0 (3)
C21—P8—C15—C20	58.5 (3)	Fe2—C29—C33—C32	58.5 (2)
C9—P8—C15—C20	170.2 (2)	C30—C29—C33—C38	172.2 (3)
Pt1—P8—C15—C20	−66.9 (3)	C34—C29—C33—C38	−0.5 (5)
C21—P8—C15—C16	−69.7 (3)	Fe2—C29—C33—C38	−128.0 (3)
C9—P8—C15—C16	42.0 (3)	C30—C29—C33—Fe2	−59.8 (2)
Pt1—P8—C15—C16	164.9 (2)	C34—C29—C33—Fe2	127.5 (3)
C20—C15—C16—C17	54.0 (4)	C31—C32—C33—C29	1.0 (4)
P8—C15—C16—C17	−177.1 (2)	C37—C32—C33—C29	176.7 (3)
C15—C16—C17—C18	−56.5 (4)	Fe2—C32—C33—C29	−60.0 (2)
C16—C17—C18—C19	58.2 (4)	C31—C32—C33—C38	−172.6 (3)
C17—C18—C19—C20	−57.8 (4)	C37—C32—C33—C38	3.1 (5)
C18—C19—C20—C15	56.8 (4)	Fe2—C32—C33—C38	126.4 (3)
C16—C15—C20—C19	−54.5 (4)	C31—C32—C33—Fe2	61.0 (2)
P8—C15—C20—C19	173.8 (2)	C37—C32—C33—Fe2	−123.3 (3)
C9—P8—C21—C22	−58.5 (3)	C27—Fe2—C33—C29	162.2 (2)
C15—P8—C21—C22	52.4 (3)	C5—Fe2—C33—C29	−14.2 (3)
Pt1—P8—C21—C22	−179.5 (2)	B3—Fe2—C33—C29	−119.7 (2)
C9—P8—C21—C26	170.3 (2)	C32—Fe2—C33—C29	119.1 (3)
C15—P8—C21—C26	−78.8 (3)	C31—Fe2—C33—C29	80.5 (2)
Pt1—P8—C21—C26	49.2 (3)	C30—Fe2—C33—C29	37.68 (19)
C26—C21—C22—C23	−56.8 (4)	Pt1—Fe2—C33—C29	−75.56 (19)
P8—C21—C22—C23	170.2 (2)	C27—Fe2—C33—C32	43.0 (2)
C21—C22—C23—C24	58.4 (4)	C5—Fe2—C33—C32	−133.3 (2)
C22—C23—C24—C25	−57.8 (4)	B3—Fe2—C33—C32	121.2 (2)
C23—C24—C25—C26	56.4 (4)	C31—Fe2—C33—C32	−38.65 (18)
C24—C25—C26—C21	−55.1 (4)	C30—Fe2—C33—C32	−81.5 (2)
C22—C21—C26—C25	55.2 (4)	C29—Fe2—C33—C32	−119.1 (3)
P8—C21—C26—C25	−170.7 (2)	Pt1—Fe2—C33—C32	165.31 (16)
C27—Fe2—C29—C33	−41.5 (4)	C27—Fe2—C33—C38	−75.4 (4)
C5—Fe2—C29—C33	171.2 (2)	C5—Fe2—C33—C38	108.2 (4)
B3—Fe2—C29—C33	65.7 (2)	B3—Fe2—C33—C38	2.7 (3)
C32—Fe2—C29—C33	−37.55 (19)	C32—Fe2—C33—C38	−118.5 (4)
C31—Fe2—C29—C33	−81.8 (2)	C31—Fe2—C33—C38	−157.1 (4)
C30—Fe2—C29—C33	−118.7 (3)	C30—Fe2—C33—C38	160.1 (4)
Pt1—Fe2—C29—C33	109.65 (18)	C29—Fe2—C33—C38	122.4 (4)
C27—Fe2—C29—C30	77.2 (4)	Pt1—Fe2—C33—C38	46.9 (3)
C5—Fe2—C29—C30	−70.2 (2)	C44—C39—C40—C41	0.3 (5)
B3—Fe2—C29—C30	−175.6 (2)	C39—C40—C41—C42	−0.6 (6)
C32—Fe2—C29—C30	81.1 (2)	C40—C41—C42—C43	0.0 (6)
C31—Fe2—C29—C30	36.84 (19)	C41—C42—C43—C44	0.8 (6)
C33—Fe2—C29—C30	118.7 (3)	C42—C43—C44—C39	−1.1 (5)
Pt1—Fe2—C29—C30	−131.67 (18)	C40—C39—C44—C43	0.5 (5)
